# Temperate tree species show cross‐tolerance to heat, drought, and late spring‐frost stress

**DOI:** 10.1111/nph.71277

**Published:** 2026-05-16

**Authors:** Norbert Kunert, Jonathan Ehrmann, Svenja Gebhard, Sophie Hofmann, Georg Zimmermann, Peter Hajek

**Affiliations:** ^1^ Functional and Tropical Plant Ecology University of Bayreuth Universitätsstraße 30 95440 Bayreuth Germany; ^2^ Disturbance Ecology and Vegetation Dynamics, Bayreuth Center of Ecology and Environmental Research (BayCEER), University of Bayreuth Universitätsstraße 30 95440 Bayreuth Germany; ^3^ Geobotany, Faculty of Biology University of Freiburg Schänzlestraße 1 Freiburg im Breisgau 79104 Germany

**Keywords:** heatwaves, late spring‐frost risk, thermal tolerance traits, tree mortality, tree resistance to climatic stress, water stress

## Abstract

Significant cross‐tolerance of leaf traits to heat, drought and late spring‐frost were found. (a) Turgor loss point vs lethal spring‐frost temperature. (b) Heat thermal threshold temperature vs lethal spring‐frost temperature. (c) Heat thermal threshold temperature vs turgor loss point.

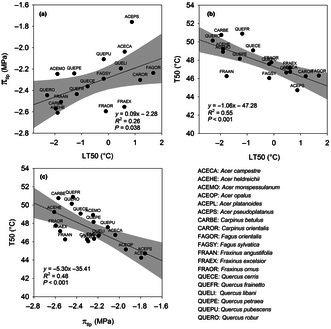

## Disclaimer

The New Phytologist Foundation remains neutral with regard to jurisdictional claims in maps and in any institutional affiliations.

## Multiple stressors in an emerging new climate

Climate change has triggered advances in spring phenology, here referred to as the trend of trees exhibiting earlier leaf unfolding, combined with a persisting risk of late spring‐frost events (Augspurger, 2009; Bigler & Bugmann, 2018; Lamichhane, 2021). Over the past six decades, the progressively earlier onset of warm spring temperatures has led to shifts in phenological timing (Zohner *et al*., 2020; Kunert & Gebhard, 2026) (Supporting Information Fig. S1). Along with an earlier leaf unfolding, the frequency of spring‐frost events has intensified by 35% over the same period in Europe and Asia (Zohner *et al*., 2020) increasing the risk of late spring‐frost damage to the young foliage (Inouye, 2000; Lamichhane, 2021). Late spring‐frost damage can substantially reduce annual productivity (Dittmar *et al*., 2006; Sangüesa‐Barreda *et al*., 2019; Vitasse *et al*., 2019), forcing trees to access carbon reserves to maintain metabolic activity and support leaf re‐flushing after such events (D'Andre*a et al*., 2019). A reduction in carbon reserves in turn will reduce a tree's capacity to deal with other stressors. Whereas extensive work is currently being conducted to understand tree responses to tolerate or acclimate to multiple stressors, particularly the combined stress induced by drought and heatwaves (e.g. Hammond *et al*., 2022), less attention has been given to the effects of late spring‐frost as third stressor.

Given the high relevance of the accelerating frequency of forest die‐off events, the identification of general adaptive trait syndromes to tolerate multiple stressors has high importance in predicting responses to ever‐emerging climate change (Niinemets, 2010). Promising physiological traits describing tree species' adaptation to excessive temperatures are measured via Chl fluorescence by assessing photochemical efficiency (*F*_v_/*F*_m_) and reflect the reaction of the most thermal sensitive components of the Photosystem II (PSII) to increasing heat exposure (Tiwari *et al*., 2021; Slot *et al*., 2021). A threshold for irreversible damage to the photochemistry is the temperature at which the quantum efficiency declines by 50% (T50) (Tiwari *et al*., 2021; Krause *et al*., 2010). T50 has been found to correlate with drought resistance traits, such as the turgor loss point (π_tlp_) in temperate tree species (Münchinger *et al*., 2023), woody vegetation across temperate biomes (Mitchell *et al*., 2025), and shrubs from semi‐arid climates (Guo *et al*., 2025). Explanations for this convergence in heat and drought resistance traits in woody species might be of an ecological and evolutionary nature as dry regions are characterized by higher temperatures (Hauck *et al*., 2025).

In contrast to the rapidly emerging knowledge on how trees deal with drought and heat, quantitative measures on late spring‐frost resistance are rare and mostly come from observation during natural frost events (Augspurger, 2009, 2013). Whereas general trends of late spring‐frost resistance can be taken from those observations, such observational studies do not give any quantitative measure of how intense the frost event needs to be to cause significant damage. To reliably predict possible damage due to persisting late spring‐frost risk, critical threshold temperatures are urgently needed. Traits representing the late spring‐frost resistance during leaf out can be assessed analogously to the trait assessment of heat tolerance described above (Kunert & Gebhard, 2026). In a broader setting, the method has been used to quantify the freezing tolerance of plant species in alpine (Taschler & Neuner, 2004; Neuner & Pramsohler, 2006; Neuner *et al*., 2013; Bucher *et al*., 2019) and boreal ecosystems (Lamontagne *et al*., 2000), but also recently to test late spring‐frost resistance of temperate trees (Kunert & Gebhard, 2026). The last‐mentioned study provided empirical evidence that the *in vitro* assessment of the late spring‐frost tolerance reflects the actual frost damage caused by a natural late spring‐frost event (Kunert & Gebhard, 2026). It remains uncovered how these late spring‐frost resistance traits relate to other traits, in particular traits with explanatory power for responses of tree species to climate change. Although the importance of heat and drought resistance traits in woody species is increasingly recognized, the relationship between these traits and other key characteristics, such as late frost resistance, is not well understood. In particular, the interaction between late frost resistance traits and other adaptive traits influencing tree species' responses to climate change is unclear. A better understanding of these relationships could provide crucial insights into how trees cope with multiple climate‐induced stresses.

Here we aim to determine whether leaf thermal tolerance and drought resistance traits expressed during the mid‐summer growth phase are associated with, or contrast with, the late frost tolerance of newly emerging spring leaves. We quantified the sensitivity of photosystem II (PSII) to temperature extremes by determining the temperature at which its maximum quantum yield decreased by 50% (T50 for heat and LT50 for cold) in 19 temperate broadleaf tree species that are native to Central Europe or that are projected to expand into the region under future climate scenarios. Additionally, we evaluated leaf‐level drought tolerance during the peak growing season by determining the leaf turgor loss point (π_tlp_). Our overarching goal was to provide an integrated assessment of potential cross‐tolerance among heat, drought and late frost stresses. Specifically, we tested the prevailing expectation that adaptation to warmer and drier climates comes at the cost of reduced frost tolerance. Species from warmer climates are expected to have lower chemical resistance and unsuitable physiological structure preventing the establishment of those species in new regions (e.g. Wen *et al*., 2018).

## Cross‐tolerance of hotter drought and late frost

**Fig. 1:**
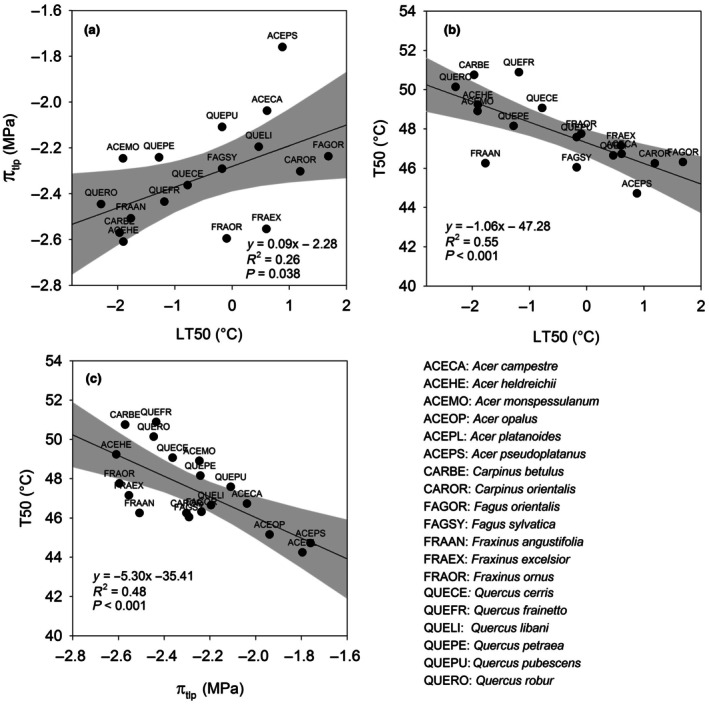
Relationships between the (a) the temperature (LT50) at which *F*_v_/*F*_m_ declines by 50% during a cooling treatment and the turgor loss point (π_tlp_), (b) the lethal temperature (LT50) during a cooling treatment and the temperature (T50) during a 30‐min heat treatment at which *F*_v_/*F*_m_ declines by 50% and (c) the temperature at which *F*_v_/*F*_m_ declines by 50% during a 30‐min heat treatment (T50) and the turgor loss point (π_tlp_).

From our measured frost, drought and heat tolerance thresholds (Table S1), we found consistent positive relationships among all three tolerance traits (Fig. 1), providing clear evidence against a trade‐off between heat, drought and late‐frost resistance (Fig. S2). A cross‐tolerance in drought resistance and frost hardiness (Fig. 1a) has so far only been connected in conifers during winter months (e.g. Blödner *et al*., 2005; Kreyling *et al*., 2012) but has not yet been described in broad‐leaved tree species, during late frost events in spring. The phenomena of cross‐tolerance in evergreen conifers have some logical explanation. During warm weather conditions in winter, stomata are opening and water starts to move through the trees. However, the trees are unable to replace the water lost through the leaves and stems as the soil water remains frozen. This winter thawing causes drought stress, and the accumulation of solutes in the leaves makes plants more resistant to winter dehydration (Bigras *et al*., 2001; Thalheimer *et al*., 2024). On the other hand, a high concentration of cytoplastic solutes avoids ice formation (Bigras *et al*., 2001; Thalheimer *et al*., 2024). Morin *et al*. (2010) show that more cold‐resistant shoot segments of oaks maintain higher carbohydrate concentrations from the beginning of dormancy until bud burst. Therefore, we assume that in our study species characterized by higher late‐frost tolerance are also characterized by a higher cytoplasmic solute concentration. Rapid osmotic adjustment in young leaves (Kunert, 2025) and higher freezing resistance in early flushing species (Vitra *et al*., 2017) support the idea that solute accumulation may link frost and drought tolerance via shared osmotic regulation mechanisms.

The observed cross‐tolerance in heat and frost tolerance (Fig. 1b) finds its explanation on a cellular level as well. Both stressors, heat, and freezing, induce similar reactions in the plant cell such as oxidative stress, signaling responses, protein denaturation, and membrane damage (Hossain *et al*., 2018). Such cross‐tolerances between heat and frost tolerance have been described as mainly induced by the priming effect of one stressor increasing the tolerance to the other stressor after being exposed to the first stressor (Walter *et al*., 2013; Antoniou *et al*., 2016). We are not aware of any priming event causing the trait correlation, however, we cannot rule out any priming capacities among species or environmental events in the recent past. Hence, we speculate that there might be common adaptive mechanisms of leaves that prevent the negative effects of temperature stress of both extremely low and high temperatures. At least for cross‐tolerance between drought and heat tolerance (Fig. 1c), we know that this cross‐tolerance is also expressed in leaf structural traits. For example, Münchinger *et al*. (2023) show that drought and heat tolerance were in the level xeromorphy of leaves, with thicker and tougher leaves showing less sensitivity. Interestingly, the slope of the relationship between T50 and π_tlp_ described by Münchinger *et al*. (2023) was like our study (slope Münchinger *et al*. (2023): −5.1; slope this study: −5.3; compare Fig. 1c), what points into the direction of a strong general relationship between these two traits even across different temperate biomes (Mitchell *et al*., 2025). Together, these findings challenge the common assumption that species adapted to warmer and drier climates necessarily sacrifice frost tolerance. Instead, our results indicate that tolerance to climatic extremes is coordinated across stress axes, suggesting the existence of an integrated stress‐resistance syndrome in temperate broadleaf trees.

## Chilling effects in temperate tree species

Our results reveal an additional, previously neglected dimension of low‐temperature stress. One‐third of the investigated tree species exhibited significant declines in PSII efficiency at temperatures above 0°C, indicating chilling injury rather than freezing damage. Physiological impairments caused by low, nonfreezing temperatures are referred to as chilling injury, in contrast to freezing below 0°C (Guo *et al*., 2018; Liu *et al*., 2018). Physiological impairment due to chilling temperatures has been described for a large variety of crop species (for an overview, see Kratsch & Wise, 2000 and Lukatkin *et al*., 2012) but has not yet been observed to affect temperate trees. However, physiological impairment caused by small temperature drops might not always be visible but can reduce productivity during the subsequent growing season (Korovin, 1968 as cited in Lukatkin *et al*., 2012). Chilling temperatures can impair multiple physiological processes, including photosynthesis, even when ice formation does not occur (Lukatkin *et al*., 2012). The significant injury usually arises at temperatures below 15°C (Raison & Lyons, 1986). In temperate regions those temperatures are common and might even occur during the peak growing season (Bramlage & Meir, 1990). Thus, plants evolved in a temperate environment should tolerate such temperature ranges. However, Bramlage & Meir (1990) also state that tolerating chilling temperatures does not mean to be immune to injury but to have a certain level of resistance to lower temperatures. In our study, the exposure to temperatures below 1.7°C inhibited a significant decline in photosynthetic quantum use efficiency and thus chilling injury. The photosystem in the chloroplast is commonly the most sensitive component of the plants showing the earliest ultrastructural changes after a chilling event (Kimball & Salisbury, 1973). The two most sensitive species were oriental beech and oriental hornbeam (LT50: 1.68°C and 1.19°C, respectively). Both species have their natural distribution in areas with lower late frost risk than in our study area. Their natural range is also characterized by higher temperatures and drier summer conditions during the summer months than our study area but did not show higher tolerance to heat and drought in their trait expression. We assume that despite the existence of hereditary the expression of those traits is more influenced by acclimation to the new site than by adaptation to the original site when species are relocated to other areas (Konôpková *et al*., 2018; Kurjak *et al*., 2019). It is noticeable that the most cross‐tolerant species tend to have the broadest natural distributions, such as pedunculate oak and European hornbeam, whereas the most sensitive species are restricted to comparatively narrow ranges in the Caucasian region. This pattern suggests that distribution range, rather than origin from climatically extreme regions *per se*, may favor the evolution of coordinated stress tolerances.

## Materials and Methods

Botanical material for this study was collected from trees growing on the campus and the Ecological Botanical Garden of the University of Bayreuth, Northern Bavaria in Germany (49.9261188° N, 11.5841229° E). The climate at Bayreuth is a marine west coast, warm summer climate. The mean annual precipitation rate is 960 mm and the mean annual temperature of 8.9°C at an elevation of 340 m above sea level (Kunert *et al*., 2024). There is a significant spring warming trend over the last 65 yr; however, spring late‐frost events do occur on a regular basis (see Fig. S1). For this study, we chose 19 tree species that cover wide environmental ranges, from the temperate and Atlantic regions of Western and Central Europe to the sub‐Mediterranean and continental areas of Southeastern Europe and Anatolia (Table S1). For all these 19 species, at least three individuals are growing on campus or the Ecological Botanical Garden. For all measurements, we collected one sun‐exposed branch from three tree individuals per species. After cutting the branches, they were placed in opaque plastic bags that contained moist tissue to avoid dehydration. The plastic bags were brought to the laboratory immediately after collecting the branches and further processed. In the laboratory, branches were recut under water to remove the embolized cut end of the branch. Branches were placed in buckets with water and covered with opaque plastic bags. After an overnight rehydration period, leaves were sampled from the rehydrated branches for further processing.

### Assessment of the heat tolerance

Botanical material for assessing heat tolerance was collected on 7 August 2023 and prepared as described above. We used an overall 40 leaves per species to establish a thermal vulnerability curve. Therefore, per branch 13–14 leaves were selected. We punched leaf discs out of the leaves with 3 cm in diameter. All leaf discs were dark‐acclimated for 30 min, and the initial maximum photosynthetic efficiency (*F*_v_/*F*_m_) was measured with a Chl fluorometer (MINI‐PAM, Walz, Effeltrich, Germany). This ensured that we used only healthy leaves with an *F*_v_/*F*_m_ between 0.83 to 0.75. The leaf discs were randomly assigned to one of eight temperature treatments between 25°C and 60°C (Kunert & Hajek, 2022). We placed the leaf discs on stiff plastic sheets, and we fixed the leaf discs with perforated medical tape (Transpore™, 3M™ GmbH, Austria) on the plastic sheets. The leaf discs were then covered with moist tissue to avoid dehydration. The plastic sheets were placed in water‐tight ziplock bags and submerged in a water bath for 30 min. Water baths were maintained with sous vide precision cookers at stable temperatures. After the temperature treatment, the leaf discs were incubated under controlled conditions (*c*. 20°C, *c*. 20 μmol m^−2^ s^−1^ light) for *c*. 24 h. The recovery of *F*_v_/*F*_m_ was measured after a 30‐min dark adaptation period.

### Turgor loss point measurements

Turgor loss point (π_tlp_) measurements were conducted with the material collected for the heat tolerance measurements on 7 August 2023. From the re‐hydrated branches, we took two leaves per branch and individual. We punched out one disc 4‐mm in diameter from each leaf. The leaf disc was wrapped in aluminum foil and shock‐frozen in liquid nitrogen (LN2). After the shock‐freezing, the discs were perforated with a dissection needle and placed in the standard 10 μl chamber well of an osmometer (VAPRO 5520; Wescor, Logan, UT, USA). The osmometer was running in auto‐repeat mode and we recorded all osmometer readings (solute concentrations) until an equilibrium was indicated. Equilibrium was defined as a 5 mmol kg^−1^ difference or less between readings. The final solute concentration value c_0_ was converted into π_tlp_ with the following equation established by Bartlett *et al*. (2012):

(Eqn 1) $$\pi_{\mathrm{tlp}}=0.832\;\left(R\times T\right)/1000\;{c_{0}}^{-0.631}$$

In the equation, *R* represents the ideal gas constant, and *T* represents the temperature in Kelvin.

### Assessment of late spring‐frost tolerance

Botanical material for the late spring‐frost tolerance was sampled from the same three trees per species as for the heat tolerance on 17 April 2024 (Kunert & Gebhard, 2026). Unfortunately, Italian maple and Norway maple were not measured during this campaign due to logistics constraints. We followed the same sampling protocol and sample preparation protocol as described above. Briefly, one sun‐exposed branch per individual was collected, recut under water, and prepared for overnight rehydration. On the next day, discs were punched out of the leaves. Where leaves were not fully unfolded, we used scissors to cut round shapes out of the leaf to induce the same injury in all leaf samples. All leaf discs were tested for healthiness with the Chl fluorometer and attached to stiff plastic sheets with perforated medical tape. The plastic sheets were covered with moist tissue. We added two drops of ‘Snomax’ solution (Snomax LLC, Englewood, CO, USA) to the tissue as an extrinsic nucleator. Snomx induces uniform ice nucleation *c*. −2°C and thus avoids treatment artifacts due to supercooling (Mittelstädt & Rudolph, 1998; Wisniewski *et al*., 2014). To treat the leaf discs with different freezing temperatures we used commercial freezers. The freezers were equipped with a heating system that allowed precise control of the temperature in each drawer. Therefore, each drawer was equipped with a ventilated heater (ventilated car heater 150 W, Shenzhen Caiqi Digital Technology, Shenzhen, China). Temperature was regulated with a microcontroller unit (Arduino Mega, Arduino, Ivrea, Italy) in combination with a thermostat. With the microcontroller, we simulated naturally occurring cooling rates of 2°C h^−1^ (Neuner *et al*., 2013) and kept the temperature in the drawer at a given target temperature for 4 h. We treated the leaf discs at eight different target temperatures at −16, −8, −4, −2, 0, +2, +4, and +10°C. After these 4 h, we warmed up the drawer with a thawing rate of 2°C h^−1^. After the treatment leaf discs were incubated under controlled conditions (15°C, *c*. 20 μmol m^−2^ s^−1^ light) for 24 h and the recovery of *F*_v_/*F*_m_ was measured after a 30‐min dark adaptation period with a Chl fluorometer (Kunert & Gebhard, 2026).

### Fitting of vulnerability curves

We fitted a log‐logistic curve for the *F*_v_/*F*_m_ response to either heat or freezing treatment as described by Kunert *et al*. (2022):

(Eqn 2) $$\frac{F_{v}}{F_{m}}=c+\frac{d-c}{1+\mathrm{Exp}\left[b\;\mathrm{Log}\left[\frac{T}{T50}\right]\right]}$$

In this equation, *T* describes the temperature, *c* is the *F*_v_/*F*_m_ of the lower plateau, and *d* is the higher plateau. *T50* (in case of freezing treatment LT50) represents the temperature at which *F*_v_/*F*_m_ declined by 50%. *b* is the slope of the curve at *T* = T50. We used the ‘modelFit’ function of the drc package in Ritz *et al*. (2016) in R to find the best fitting model. We used Akaike's information criterion to decide between models. Different measures (T50 and LT50) were extracted using the ‘ED’ from the fitted curves. All presented means from these curves are with ±SE. We performed the data analysis using the R program, v.4.4.0 (R Core Team, 2024).

### Statistical analysis

Pearson's product–moment correlation coefficient was utilized to measure the bivariate relationships between T50, π_tlp_ and LT50. The assumptions of normality and homogeneity of variance were tested using Shapiro–Wilk and Levene's test, respectively. To evaluate multivariate relationships between the three physiological tolerance traits (T50, π_tlp_, and LT50), a PCA was performed. Before analysis, data were centered and scaled to ensure equal weighting of all variables. Italian maple and Norway maple were removed from the analysis, since their LT50 value could not have been measured as mentioned above, resulting in 17 species for the multivariate analysis (*n* = 17). The PCA was conducted using the prcomp function in R Core Team (2024). Results were then visualized as a distance biplot using the factoextra package (Kassambara & Mundt, 2020).

## Competing interests

None declared.

## Author contributions

NK, JE and PH designed the study. NK, SG, SH and GZ collected the data. NK and JE performed the statistical analysis. NK wrote the first version of the manuscript. All authors contributed to drafting the final version of the manuscript.

## Supporting information


**Fig. S1** Changes in spring temperatures indicated by the first warm day of the year and the last cold day since 1960 until 2025 (DWD, 2025; station 11454).
**Fig. S2** PCA of the three investigated physiological tolerance traits (T50, π_tlp_ and LT50).
**Table S1** Summary of the measured traits for the 19 tree species.
Please note: Wiley is not responsible for the content or functionality of any Supporting Information supplied by the authors. Any queries (other than missing material) should be directed to the *New Phytologist* Central Office.

## Data Availability

The data are available at doi: 10.5281/zenodo.20035030.
