# Supplementary material for: Temperate tree species show cross‐tolerance to heat, drought, and late spring‐frost stress
Source: New Phytol. 2026 May 16;251(4):1625–30. doi: 10.1111/nph.71277 (PMC13373860; doi:10.1111/nph.71277)
Supplement: Supplementary file 1 — Fig. S1 Changes in spring temperatures indicated by the first warm day of the year and the last cold day since 1960 until 2025 (DWD, 2025; station 11454). Fig. S2 PCA of the three investigated physiological tolerance traits (T50, πtlp and LT50). Table S1 Summary of the measured traits for the 19 tree species. Please note: Wiley is not responsible for the content or functionality of any Supporting Information supplied by the authors. Any queries (other than missing material) should be directed to the New Phytologist Central Office. [file NPH-251-1625-s001.pdf]

New Phytologist Supporting Information

Article title: Temperate tree species show cross-tolerance to heat, drought, and late spring-frost stress

Authors: Norbert Kunert, Jonathan Ehrmann, Svenja Gebhard, Sophie Hofmann, Georg Zimmermann, Peter Hajek

Article acceptance date: 30 April 2026

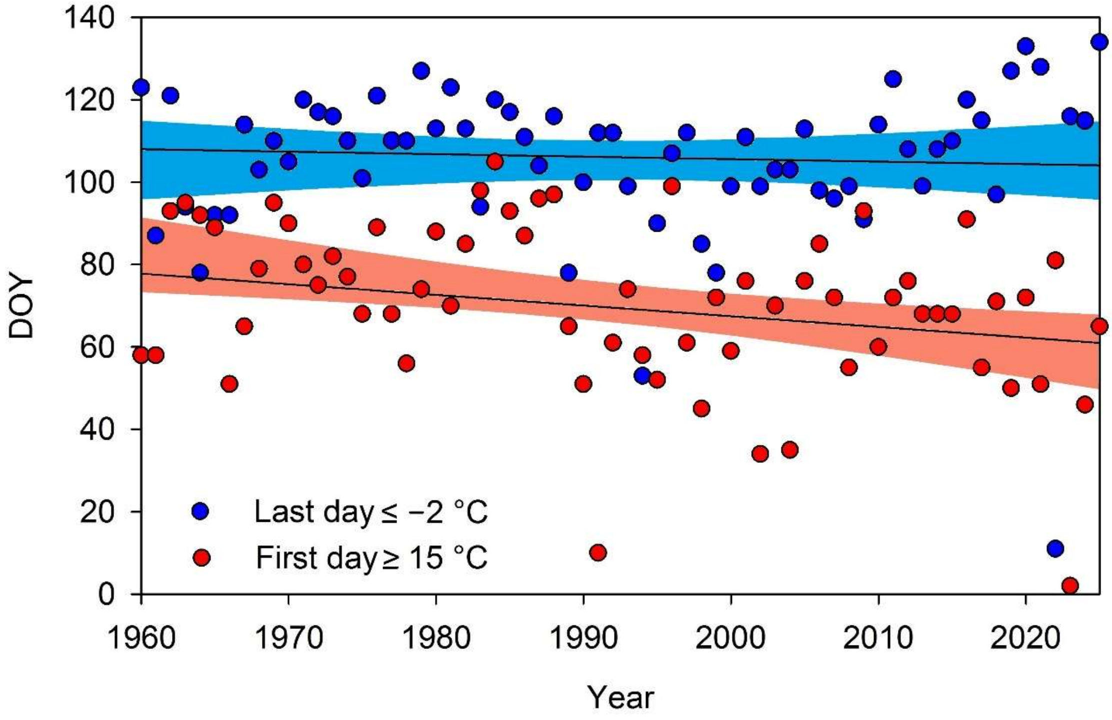

Figure S1: Changes in spring temperatures indicated by the first warm day of the year (DOY; red circles: maximum daily temperature ≥ 15°C; y = -0.259x + 586.28, R^2 = 0.07, p = 0.027) and the last cold day (blue circles: minimum daily temperatures ≤ -2°C; y = -0.061x + 227.05, R^2 = 0.005, p = 0.571) since 1960 until 2025 (DWD, 2025; station 11454). The shaded area indicates the 95% confidence interval of the linear regression.

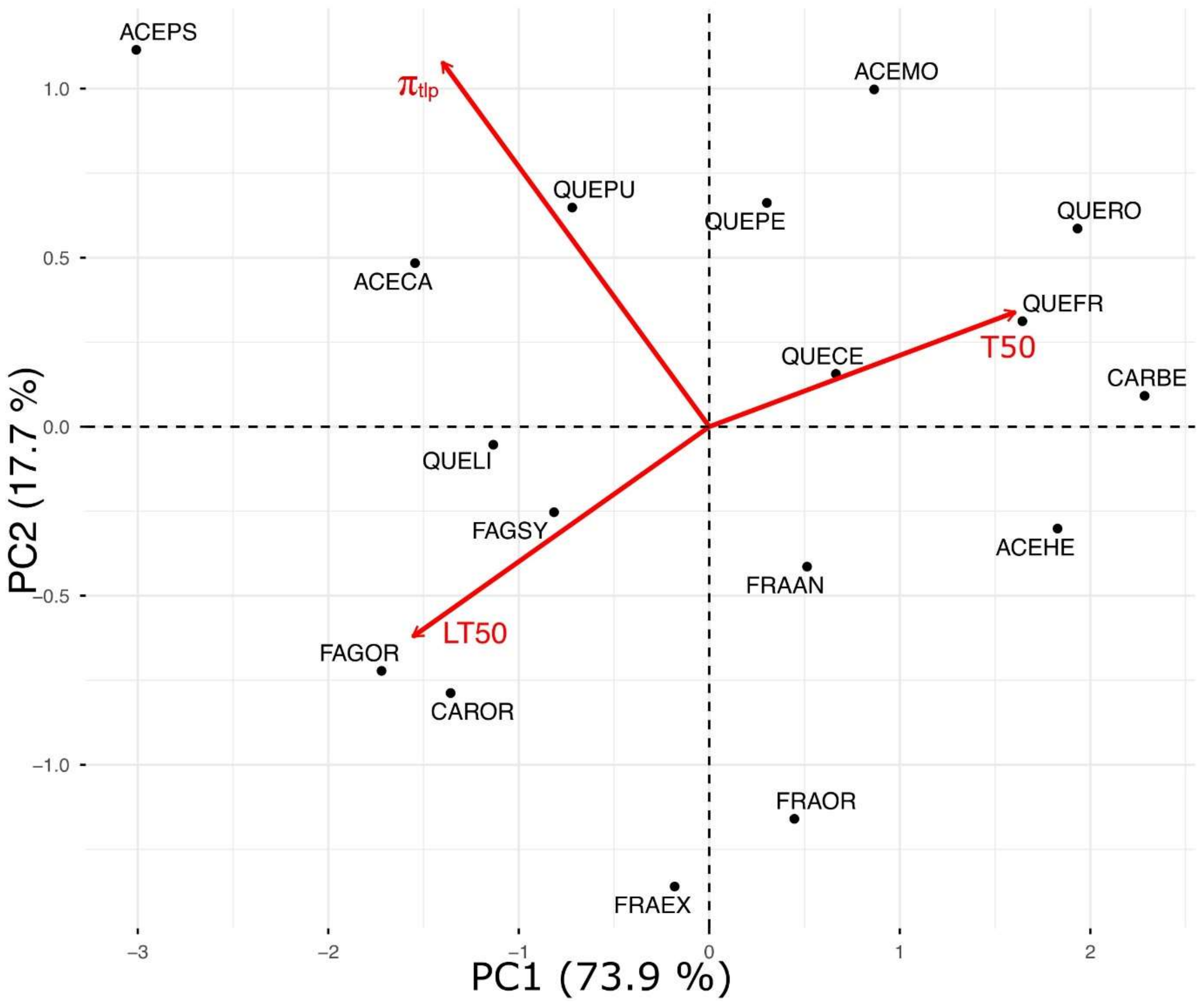

Figure S2: Principal component analysis (PCA) of the three investigated physiological tolerance traits (T50, π_{tlp} and LT50).

Table S1: Summary of the measured traits for the 19 tree species. Heat tolerance, given as temperature at which photosynthetic quantum use efficiency is at 50% (T50) of the maximum, the turgor loss point (π_{tlp}) and temperature at which 50% of the quantum use efficiency remains after a freezing treatment (LT50).

Common name	Binomial name	Family	Species abbreviation	T50		π_{tlp}		LT50	
				Mean	(SE)	Mean	(SD)	Mean	(SE)
Field maple	*Acer campestre* L.	Sapindaceae	ACECA	46.7	(2.6)	-2.04	(0.10)	0.61	(1.06)
Balkan maple	*Acer heldreichii* Orph. Ex Boiss	Sapindaceae	ACEHE	49.2	(0.4)	-2.61	(0.09)	-1.90	(0.65)
Montpellier maple	*Acer monspessulanum* L.	Sapindaceae	ACEMO	48.9	(1.0)	-2.25	(0.08)	-1.91	(0.32)
Italian maple	*Acer opalus* Mill.	Sapindaceae	ACEOP	45.2	(0.1)	-1.94	(0.13)	NA	(NA)
Norway maple	*Acer platanoides* L.	Sapindaceae	ACEPL	44.2	(1.8)	-1.80	(0.15)	NA	(NA)
Sycamore maple	*Acer pseudoplatanus* L.	Sapindaceae	ACEPS	44.7	(0.6)	-1.76	(0.20)	0.88	(0.75)
European hornbeam	*Carpinus betulus* L.	Betulaceae	CARBE	50.8	(0.8)	-2.57	(0.13)	-1.97	(0.28)
Oriental hornbeam	*Carpinus orientalis* Mill.	Betulaceae	CAROR	46.2	(1.0)	-2.30	(0.13)	1.19	(0.49)
Oriental beech	*Fagus orientalis* Lipsky	Fagaceae	FAGOR	46.3	(0.7)	-2.24	(0.15)	1.68	(0.23)
European beech	*Fagus sylvatica* L.	Fagaceae	FAGSY	46.0	(1.3)	-2.29	(0.07)	-0.17	(1.78)
Narrow-leaved ash	*Fraxinus angustifolia* Vahl	Oleaceae	FRAAN	46.3	(0.6)	-2.51	(0.10)	-1.77	(0.08)
European ash	*Fraxinus excelsior* L.	Oleaceae	FRAEX	47.1	(0.7)	-2.55	(0.16)	0.60	(0.26)
Manna ash	*Fraxinus ornus* L.	Oleaceae	FRAOR	47.8	(0.4)	-2.60	(0.15)	-0.09	(0.27)
Austrian oak	*Quercus cerris* L.	Fagaceae	QUECE	49.1	(0.9)	-2.36	(0.14)	-0.78	(0.37)
Hungarian oak	*Quercus frainetto* Ten.	Fagaceae	QUEFR	50.9	(0.5)	-2.43	(0.08)	-1.18	(0.10)
Lebanon oak	*Quercus libani* G.Olivier	Fagaceae	QUELI	46.6	(1.5)	-2.19	(0.10)	0.47	(0.50)
Sessile oak	*Quercus petraea* (Matt.) Liebl.	Fagaceae	QUEPE	48.1	(2.3)	-2.24	(0.10)	-1.28	(0.81)
Downy oak	*Quercus pubescens* Willd.	Fagaceae	QUEPU	47.6	(0.8)	-2.11	(0.11)	-0.17	(0.72)
Pedunculate oak	*Quercus robur* L.	Fagaceae	QUERO	50.1	(3.0)	-2.45	(0.09)	-2.29	(1.11)
